# Admission APRI and Early Mortality in ICU Patients with Sepsis: A Retrospective Cohort Study

**DOI:** 10.3390/microorganisms14091922

**Published:** 2026-08-31

**Authors:** Ibrahim A. Alqarni, Zeina S. Alkudmani, Naelah A. Alghamdi, Hamad Almatrudi, Muath Alsaidan, Yazeed Alshuweishi

**Affiliations:** 1 Department of Clinical Laboratory Sciences, College of Applied Medical Sciences, King Saud University, Riyadh 12372, Saudi Arabia; 2 Department of Physiology, College of Medicine, King Saud University, Riyadh 11461, Saudi Arabia; 3 Department of Pathology and Laboratory Medicine, Medical Services, Ministry of Interior, Riyadh 11481, Saudi Arabia; 4 Department of Family and Community Medicine, College of Medicine, King Saud University, Riyadh 12372, Saudi Arabia

**Keywords:** sepsis, intensive care unit, aspartate aminotransferase-to-platelet ratio index (APRI), early mortality, survival analysis

## Abstract

Early identification of ICU patients with sepsis who are at risk of early death remains clinically important. The aspartate aminotransferase-to-platelet ratio index (APRI) is a simple laboratory-derived index, but its clinical value for very early mortality in adult ICU patients with sepsis remains insufficiently characterized. This study evaluated the association between admission APRI and short-term mortality in ICU patients with sepsis. This retrospective cohort study included 266 adults admitted to the ICU with sepsis. APRI was calculated from AST and platelet measurements obtained within 24 h of admission. Patients were categorized according to mortality timing. Associations with 7-day mortality were evaluated using logistic regression adjusted for age, sex, and comorbidity status. ROC and survival analyses were also performed. Of 266 patients, 32 died within 7 days, 88 between 8 and 28 days, and 72 after 28 days, and 74 survived. APRI was highest among patients who died within 7 days (median 1.919, IQR 0.631–5.009) compared with survivors (0.472, IQR 0.192–1.531; *p* = 0.0056). Higher log-transformed APRI remained associated with 7-day mortality after adjustment for age, sex, and comorbidity status (OR = 2.77, 95% CI 1.57–5.01; *p* = 0.0004). APRI showed modest discrimination for 7-day mortality (AUC = 0.687, 95% CI 0.580–0.793) and lower discrimination for 28-day mortality (AUC = 0.623, 95% CI 0.555–0.690). Elevated APRI was also associated with reduced short-term survival. Elevated admission APRI is independently associated with early mortality and reduced short-term survival in ICU patients with sepsis. However, its modest discrimination supports its use as an adjunct to established clinical risk assessment tools.

## 1. Introduction

Sepsis is a complex clinical condition that develops when the body response to infection becomes abnormal and poorly regulated leading to systemic inflammation, progressive organ dysfunction and potentially fatal outcomes [1]. Despite advances in antimicrobial therapy and critical care, sepsis remains a major cause of mortality among critically ill patients, particularly when progression to septic shock results in profound circulatory and metabolic disturbances and multi-organ failure [2,3]. In addition to its high mortality, sepsis is associated with prolonged hospitalization, increased healthcare costs, and long-term physical and functional impairment among survivors [4]. Because patient outcomes are strongly affected by the timing of the diagnosis and initiation of appropriate treatments, identifying individuals at high risk of adverse outcomes as early as possible remains a major priority in critical care medicine.

Accurate risk stratification is fundamental to the management of critically ill patients, as it enables clinicals to identify those at greatest risk of deterioration and improve clinical decision making. An effective prognostic model should rely on objective, readily available, and consistently measurable clinical and laboratory variables that can be obtained during routine patients care. Several severity scoring systems, including the acute physiology and chronic health evaluation (APACHE), and the sequential organ failure assessment (SOFA), are widely used to estimate disease severity and predict outcomes as in-hospital mortality [5,6,7,8]. Although these tools have demonstrated robust predictive performance, most were done using cohorts from well-researched healthcare system. Therefore, their predictive value may vary in other clinical settings where patients’ characteristics, healthcare infrastructure and data availability differ substantially. This prompted increasing interest in identifying simpler, more accessible biomarkers and developing validated prognostic models that can improve risk prediction across diverse healthcare environments. Recent evidence has demonstrated that several complete blood count (CBC)-derived inflammatory indices, including the neutrophil-to-lymphocyte ratio (NLR), Systemic Inflammatory Response Index (SIRI), Systemic Immune-Inflammation Index (SII), and Aggregate Index of Systemic Inflammation (AISI), are associated with mortality in patients with sepsis [9,10,11,12]. A recent systematic review and meta-analysis involving more than 23,000 patients confirmed that elevated NLR is significantly associated with increased mortality, while large multicentre cohort studies have shown that multiple inflammation-derived hematological indices provide independent prognostic information despite only moderate predictive performance [13,14]. Collectively, these findings suggest that routinely available laboratory indices may complement conventional severity scores; however, most investigated biomarkers primarily reflect systemic inflammatory cell responses, with comparatively little attention given to biomarkers representing organ-specific injury.

In this context, the Aspartate Aminotransferase-to-Platelet Ratio Index (APRI) may be particularly relevant because it integrates two abnormalities commonly observed during severe systemic illness, hepatocellular injury and thrombocytopenia. During sepsis, hepatocellular injury, tissue hypoperfusion, and systemic inflammation may contribute to increased AST levels, while platelet consumption, coagulation activation, and endothelial dysfunction may contribute to reduced platelet counts [15,16,17]. By combining these two parameters, APRI may therefore capture aspects of early systemic and organ dysfunction occurring during the acute phase of sepsis. Although originally developed as a non-invasive marker of hepatic fibrosis, APRI has increasingly been investigated as a marker of adverse outcomes in acute and critical illness. Emerging evidence supports the clinical relevance of APRI beyond chronic liver disease. In patients with sepsis, higher APRI has been associated with sepsis-associated liver injury, liver dysfunction, greater disease severity, and poorer clinical outcomes in both pediatric and adult populations [18,19]. Its prognostic relevance has also been reported in other critically ill populations. In hospitalized patients with COVID-19, elevated APRI independently predicted both short- and long-term mortality, whereas in patients with heat stroke, increased APRI was associated with significantly reduced survival and independently predicted early mortality [20,21]. Similarly, studies in patients with cardiogenic shock have demonstrated that elevated APRI is associated with increased in-hospital mortality, further supporting its value as a marker of adverse outcomes in critical illness [22]. Collectively, these findings suggest that APRI is not merely a marker of chronic liver disease but rather a readily available biomarker that captures key pathophysiological processes underlying acute critical illness.

However, APRI has been studied primarily in relation to sepsis-associated liver injury and broader clinical outcomes, while evidence regarding its association with very early mortality following ICU admission remains limited. Few studies have specifically examined the clinical value of admission APRI for early mortality in adult ICU patients with sepsis. Therefore, the aim of this study was to determine whether admission APRI is independently associated with early mortality and to assess its utility as a simple, inexpensive, and readily available biomarker for early risk stratification in critically ill patients with sepsis.

## 2. Materials and Methods

### 2.1. Study Population

A retrospective observational study was conducted at King Saud University Medical City (KSUMC) using electronic medical records and the laboratory information system. Adult patients (≥18 years) admitted to the ICU between 2018 and 2015 with a documented clinical diagnosis of sepsis in the electronic medical record were eligible. Sepsis cases were identified retrospectively based on the clinical diagnosis documented by the treating team rather than through independent retrospective application of Sepsis-3 criteria. In addition, SOFA and APACHE II scores were not consistently available, preventing adjustment for established measures of illness severity. Therefore, residual confounding by sepsis severity cannot be excluded, and the prognostic association observed for APRI should be interpreted accordingly. Collected data included baseline demographics, comorbidities, CBC parameters, biochemical and renal markers, lipid profile, and procalcitonin levels. Data were deidentified, securely stored, and analyzed using appropriate statistical methods.

**Figure 1:**
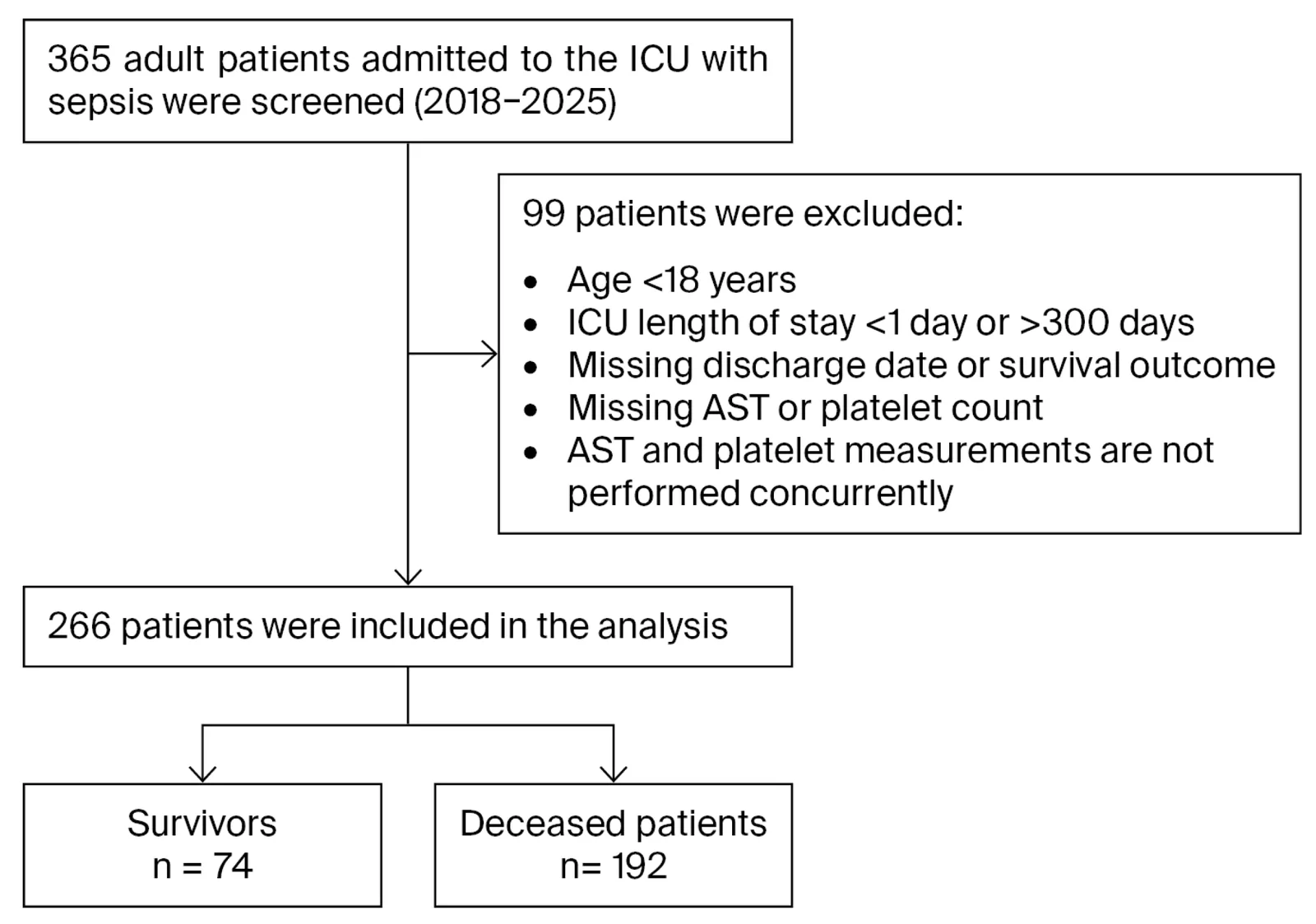
Flow diagram of patient selection and study enrollment.

As shown in Figure 1, only adult patients admitted to the ICU with a diagnosis of sepsis were included. Patients without a recorded discharge date were excluded from the analysis. Patients with an ICU length of stay of less than 1 day, or greater than 300 days, were also excluded. The final analytical cohort was defined by the availability of the variables required to calculate admission APRI and determine mortality status and timing. For other variables with incomplete data, no imputation was performed, and analyses were conducted using available cases. Accordingly, sample sizes varied across analyses according to data availability, with available sample sizes for variables with missing observations reported in the corresponding tables.

### 2.2. Laboratory Test Measurements

Laboratory investigations were performed within the first 24 h of ICU admission using the fully automated laboratory system at King Saud University Medical City. A comprehensive panel of laboratory parameters was analyzed, including complete blood count (CBC), liver function tests, inflammatory markers, and routine biochemical indices. Biochemical analyses were performed using the cobas c 702 chemistry analyzer (Roche Diagnostics, Mannheim, Germany), while hematological parameters were measured using the Sysmex XN-10/XN-20 hematology analyzers (Sysmex Corporation, Kobe, Japan). Both platforms were fully integrated within the cobas^®^ 8100 Total Laboratory Automation (TLA) system (Roche Diagnostics), ensuring standardized sample processing and analytical workflow.

The AST-to-Platelet Ratio Index (APRI) was calculated from the first AST and platelet measurements obtained within 24 h of ICU admission using the following formula [23,24]: APRI = [(AST/AST upper limit of normal) × 100]/platelet count. AST was expressed in U/L, with 40 U/L used as the upper limit of normal, and platelet count was expressed as ×10^9^/L. For categorical analyses, admission APRI was classified using a predefined threshold of 0.5, selected a priori based on its conventional use as a reference threshold in the assessment of hepatic fibrosis [25]. In the present study, this threshold was used for exploratory stratification of patients into lower (<0.5) and elevated (≥0.5) APRI groups and was not considered a validated cut-off for mortality prediction in sepsis. Separately, outcome-specific cut-off values for 7-day and 28-day mortality were derived from ROC curve analyses based on the optimal balance between sensitivity and specificity.

### 2.3. Data Collection

The first laboratory reading obtained within the first 24 h of ICU admission was collected for each patient. All data, including baseline demographic information (age, sex, nationality, and BMI), comorbidities (diabetes mellitus, cardiovascular disease, chronic kidney disease, and cancer), length of ICU stay, admission and discharge dates, laboratory results, and patient outcomes, were obtained electronically from the hospital’s Information Technology Department. Length of ICU stay was calculated in days as the interval between the date of ICU admission and the date of discharge.

### 2.4. Statistical Analysis

Continuous variables were assessed for normality and are presented as median with interquartile range (IQR), whereas categorical variables are presented as frequencies and percentages. Comparisons between two groups were performed using the Mann–Whitney U test for continuous variables and the Pearson χ^2^ test for categorical variables. Comparisons across multiple mortality groups (survivors, late mortality, 8–28-day mortality, and 7-day mortality) were performed using the Kruskal–Wallis test followed by Dunn’s multiple-comparison test. Associations between admission APRI and continuous clinical and laboratory variables were assessed using Spearman’s rank correlation analysis. The prevalence of elevated APRI across mortality groups was compared using the Pearson χ^2^ test, with the Cochran–Armitage test for trend and Fisher’s exact test for pairwise comparisons.

The association between admission log-transformed APRI and 7-day mortality was evaluated using logistic regression in unadjusted and multivariable models adjusted for age, sex, and comorbidities. Because the APRI distribution was markedly right-skewed, a sensitivity analysis was performed to assess the potential influence of extreme upper values on the association with 7-day mortality. Extreme observations were defined a priori using the upper outer fence (Q3 + 3 × IQR), corresponding to APRI > 7.23. The primary logistic regression analyses were repeated after exclusion of these observations, while all observations were retained in the primary analyses. Moreover, Kaplan–Meier survival curves were generated for 7-day and 28-day mortality and compared using the log-rank (Mantel–Cox) and Gehan–Breslow–Wilcoxon tests. Multivariable Cox proportional hazards regression was performed to estimate hazard ratios (HRs) for 7-day and 28-day mortality after adjustment for age, sex, and comorbidities. Receiver operating characteristic (ROC) curve analysis was used to assess the discriminatory performance of admission APRI, with the area under the curve (AUC), sensitivity and specificity reported. A two-sided *p* < 0.05 was considered statistically significant.

## 3. Results

### 3.1. Admission Clinical and Laboratory Characteristics

Among the 266 ICU patients with sepsis included in this study, 192 (72.2%) died and 74 (27.8%) survived (Table 1). Age, sex distribution, ICU length of stay, and inflammatory markers (CRP, procalcitonin, WBC) did not differ significantly between survivors and non-survivors. Non-survivors had significantly higher BMI (28.8 vs. 25.6 kg/m^2^, *p* = 0.016), significantly lower serum albumin (26.2 vs. 29.7 g/L, *p* = 0.005), lower RBC counts (3.70 vs. 4.05 × 10^12^/L, *p* = 0.007), and lower hemoglobin levels (102.0 vs. 110.0 g/L, *p* = 0.018) compared with survivors. AST, ALT, total bilirubin, and platelet counts did not differ significantly between the two groups.

### 3.2. Admission APRI According to Mortality Groups

**Figure 2:**
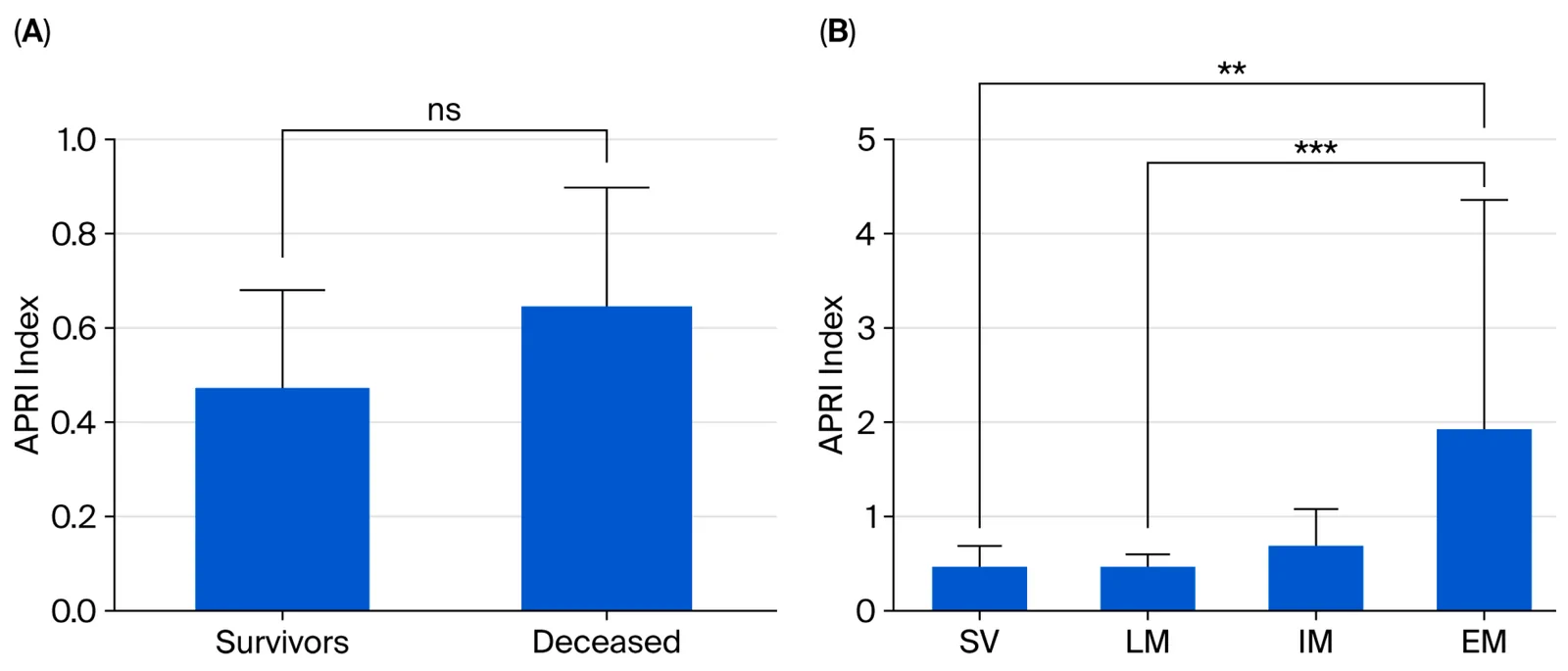
Admission APRI according to mortality status in ICU patients with sepsis. (**A**) Comparison of admission APRI between overall survivors and non-survivors. No significant difference in admission APRI was observed between the two groups (Mann–Whitney U test, *p* = 0.1806). (**B**) Admission APRI stratified according to timing of death: SV: survivors; LM: late mortality; IM: intermediate mortality; and EM: early mortality. Data are presented as median with interquartile range (IQR). Statistical comparisons were performed using the Kruskal–Wallis test followed by Dunn’s multiple-comparison post hoc test. ** = *p* < 0.01; *** *p* < 0.001; ns, not significant.

Among the 266 ICU patients with sepsis, the median admission APRI was numerically higher in patients who died than in survivors (0.645 IQR: 0.239–2.093) vs. (0.472 IQR: 0.192–1.531), respectively. However, this difference did not reach statistical significance (Mann–Whitney U test, *p* = 0.181). Admission APRI showed substantial variability in both groups, with values ranging from 0.032 to 92.59 among deceased patients and from 0.039 to 36.94 among survivors (Figure 2A). In contrast, admission APRI differed significantly across mortality timing groups (Figure 2B, Kruskal–Wallis test, *p* < 0.001). Patients who died within the first 7 days after ICU admission (early mortality) exhibited the highest admission APRI (median 1.919, IQR:0.631–5.009), compared with patients who died between 8 and 28 days (median 0.686, IQR: 0.252–2.030), those who died after 28 days (median 0.468, IQR: 0.186–1.006), and survivors (median 0.472, IQR: 0.192–1.531). Post hoc Dunn’s multiple-comparison analysis demonstrated that admission APRI was significantly higher in the early mortality group than in survivors (*p* = 0.0056) and the late mortality group (*p* = 0.0007), whereas no significant differences were observed between the early and intermediate mortality groups (*p* = 0.1615) or among the remaining pairwise comparisons (all *p* > 0.05).

### 3.3. Correlation Between Admission APRI and Laboratory Characteristics in ICU Patients with Sepsis

Spearman correlation analysis was performed to evaluate the relationship between admission APRI and selected clinical and laboratory parameters in ICU patients with sepsis (Table 2). Admission APRI demonstrated a moderate positive correlation with total bilirubin (r = 0.432, *p* < 0.001) and a weak-to-moderate positive correlation with indirect bilirubin (r = 0.279, *p* < 0.001). Weak positive correlations were also observed with blood urea nitrogen (r = 0.128, *p* = 0.037) and phosphorus (r = 0.169, *p* = 0.006), whereas a weak negative correlation was identified with serum albumin (r = −0.127, *p* = 0.038). No significant correlations were found between APRI and inflammatory or hematological parameters, including white blood cell count, neutrophil count, lymphocyte count, C-reactive protein, procalcitonin, hemoglobin, hematocrit, sodium, osmolality, or total protein (all *p* > 0.05).

### 3.4. Prevalence of Elevated Admission APRI According to Mortality Status

As shown in Table 3, the prevalence of elevated admission APRI differed significantly across mortality groups (Pearson χ^2^ = 10.84, df = 3, *p* = 0.0126). Patients who died within 7 days had the highest prevalence of elevated APRI (78.1%), followed by those who died between 8 and 28 days (60.2%), whereas patients with late mortality and survivors demonstrated lower prevalences (47.2% and 48.6%, respectively). A significant linear trend was observed across the ordered mortality groups (Chi-square test for trend: χ^2^ = 8.449, df = 1, *p* = 0.0037), indicating that the prevalence of elevated APRI increased with earlier mortality. Pairwise comparisons further demonstrated that the prevalence of elevated APRI was significantly higher among patients with 7-day mortality than among survivors (*p* = 0.0055) and patients with late mortality (*p* = 0.0049), whereas no significant difference was observed between the 7-day and 28-day mortality groups (*p* = 0.0849).

Pairwise comparisons with the 7-day mortality group were performed using the two-sided Fisher’s exact test. Overall comparison was performed using the Pearson Chi-square test. Trend analysis was performed using the Cochran–Armitage Chi-square test for trend.

### 3.5. Adjusted Associations Admission APRI and 7-Day Mortality

A series of logistic regression analyses were performed to examine the association between admission log-transformed APRI and 7-day mortality among ICU patients (Table 4). In the unadjusted model, admission APRI was significantly associated with increased odds of early mortality (OR = 2.74, 95% CI: 1.58–4.87, *p* = 0.0003). This association remained significant after adjustment for age and sex (Model 1: OR = 2.74, 95% CI: 1.56–4.95, *p* = 0.0005) and after further adjustment for the presence of one or more major comorbidities (Model 2: OR = 2.77, 95% CI: 1.57–5.01, *p* = 0.0004). Age, sex, and comorbidity burden were not independently associated with early mortality in the adjusted models (all *p* > 0.05). Because the APRI distribution was markedly right-skewed, a sensitivity analysis was performed to assess the influence of extreme upper values. Extreme observations were objectively defined using the upper outer fence (Q3 + 3 × IQR), corresponding to APRI > 7.23. Eighteen patients met this criterion and were excluded from the sensitivity analysis. After exclusion of these observations, log-transformed APRI remained significantly associated with 7-day mortality in the unadjusted model (OR = 2.64, 95% CI 1.23–5.88; *p* = 0.013) and after adjustment for age, sex, and comorbidity status (adjusted OR = 2.45, 95% CI 1.12–5.55; *p* = 0.025). The adjusted effect estimate was modestly attenuated compared with the primary analysis (OR = 2.77, 95% CI 1.57–5.01; *p* = 0.0004), while the direction and statistical significance of the association were preserved.

### 3.6. Diagnostic Performance of Admission APRI for Early and Short-Term Mortality

Receiver operating characteristic (ROC) curve analysis demonstrated that admission APRI was significantly associated with both 7-day and 28-day mortality (Table 5). Admission APRI showed modest discriminatory performance for 7-day mortality, with an AUC of 0.687 (95% CI 0.580–0.793; *p* < 0.001). The optimal APRI cut-off value was 1.015, corresponding to a sensitivity of 68.4% and a specificity of 65.6%. For 28-day mortality, admission APRI also demonstrated significant, although lower, discriminative ability (AUC = 0.623, 95% CI 0.555–0.690, *p* < 0.001). Although discrimination was greater for 7-day than for 28-day mortality, the magnitude of the AUC indicates that APRI alone provides limited discriminatory ability. Furthermore, ROC-derived optimal cut-off value was 0.627, yielding a sensitivity of 62.3% and a specificity of 60.8%. These outcome-specific thresholds were derived empirically from the present cohort and should be distinguished from the predefined APRI threshold of 0.5 used for categorical analyses.

### 3.7. Kaplan–Meier Survival Analysis According to Admission APRI

**Figure 3:**
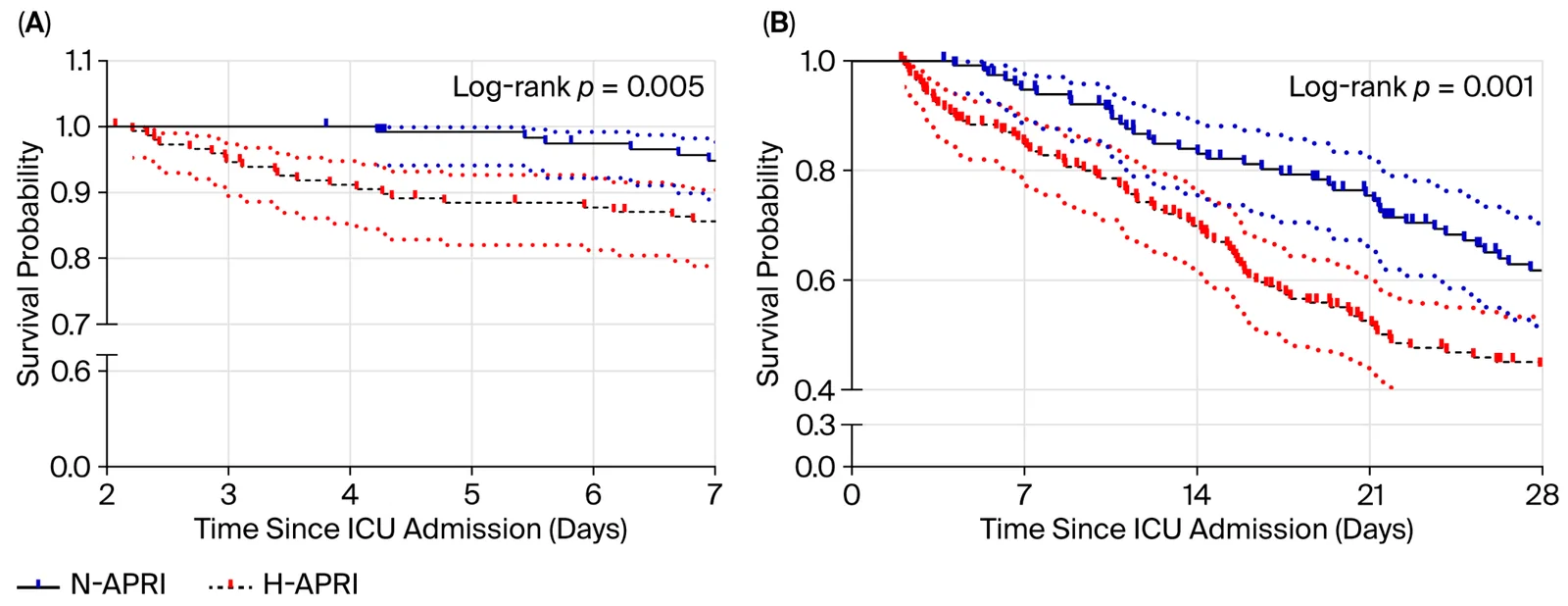
Kaplan–Meier survival curves according to admission APRI among ICU patients with sepsis. (**A**) Seven-day survival stratified by admission APRI category. Patients with elevated APRI demonstrated significantly lower 7-day survival than those with normal APRI (log-rank χ^2^ = 7.77, *p* = 0.0053; Gehan–Breslow–Wilcoxon χ^2^ = 8.24, *p* = 0.0041). (**B**) Twenty-eight-day survival stratified by admission APRI category. Patients with elevated APRI also exhibited significantly lower 28-day survival compared with those with normal APRI (log-rank χ^2^ = 10.45, *p* = 0.0012; Gehan–Breslow–Wilcoxon χ^2^ = 12.95, *p* = 0.0003). Shaded regions represent the 95% confidence intervals, and tick marks indicate censored observations.

Kaplan–Meier survival analysis demonstrated significantly lower survival among patients with elevated admission APRI compared with those with normal APRI (Figure 3). During the first 7 days following ICU admission, patients with elevated APRI had a significantly higher hazard of mortality (Figure 3A; log-rank HR = 3.10, 95% CI 1.55–6.21; log-rank χ^2^ = 7.77, *p* = 0.0053). Similar findings were observed using the Gehan–Breslow–Wilcoxon test (χ^2^ = 8.24, *p* = 0.0041), indicating that survival differences were most pronounced during the early phase following ICU admission. The association remained significant over the 28-day follow-up period. Patients with elevated admission APRI exhibited a significantly higher hazard of 28-day mortality than those with normal APRI (Figure 3B; log-rank HR = 1.84, 95% CI 1.28–2.63; log-rank χ^2^ = 10.45, *p* = 0.0012). The Gehan–Breslow–Wilcoxon test likewise demonstrated a significant difference between the survival curves (χ^2^ = 12.95, *p* = 0.0003), supporting an association between elevated admission APRI and reduced short-term survival among ICU patients with sepsis.

### 3.8. Adjusted Cox Proportional Hazards Regression Analysis of 7-Day and 28-Day Mortality

Multivariable Cox proportional hazards regression was performed to evaluate the association between admission log-transformed APRI and time to death after adjustment for age, sex, and the presence of comorbidities (Table 6). Higher admission log (APRI) was independently associated with an increased hazard of both 7-day mortality (HR = 2.59, 95% CI 1.55–4.32, *p* < 0.001) and 28-day mortality (HR = 1.91, 95% CI 1.44–2.54, *p* < 0.001). Age was not associated with 7-day mortality (HR = 1.01, 95% CI 0.99–1.03, *p* = 0.398), but was independently associated with 28-day mortality, with each one-year increase in age corresponding to a 1.8% higher hazard of death (HR = 1.02, 95% CI 1.01–1.03, *p* = 0.003). Sex and the presence of comorbidities were not independently associated with either 7-day or 28-day mortality (all *p* > 0.05). Overall, higher admission APRI was associated with an increased hazard of both early and short-term mortality in ICU patients with sepsis.

## 4. Discussion

The present study evaluated the prognostic value of admission AST-to-Platelet Ratio Index (APRI) in ICU patients with sepsis and demonstrated several important findings. The principal finding was that elevated admission APRI was significantly associated with early (7-day) mortality and remained an independent predictor after adjustment for age, sex, and comorbidities. Kaplan–Meier survival analysis further demonstrated significantly lower survival probabilities among patients with elevated APRI, while Cox proportional hazards regression confirmed that a higher admission APRI was independently associated with an increased hazard of early death. In addition, admission APRI showed moderate discriminatory ability for identifying patients at risk of early mortality and differed significantly across mortality groups, with the highest values observed among patients who died within the first week of ICU admission. Conversely, admission APRI was not associated with overall ICU mortality, suggesting that its prognostic utility is greatest during the early phase of sepsis rather than throughout prolonged critical illness. Finally, APRI demonstrated significant correlations with serum bilirubin, albumin, blood urea nitrogen, and phosphorus, suggesting that APRI may reflect abnormalities related to hepatic and systemic dysfunction, although these correlations do not establish the underlying mechanism. Collectively, these findings indicate that APRI is an inexpensive and readily available biomarker that may complement conventional clinical assessment for the early identification of high-risk patients with sepsis, particularly those at risk of early mortality.

The biological mechanisms that may underlie the association between elevated APRI and mortality are likely multifactorial. APRI integrates two parameters that are frequently altered during severe systemic illness: AST and platelet count. Elevated AST may reflect hepatocellular injury occurring in the setting of sepsis-associated liver dysfunction, hepatic hypoperfusion, cytokine-mediated injury, and microcirculatory disturbances [26]. During sepsis, impaired hepatic perfusion, mitochondrial dysfunction, and excessive inflammatory signaling may contribute to hepatocellular injury and subsequent AST release into the circulation [27]. However, AST is not liver-specific, and elevations may also arise from injury to other tissues during critical illness. Simultaneously, thrombocytopenia in sepsis may result from several mechanisms, including increased platelet consumption, activation of the coagulation cascade, endothelial injury, immune-mediated destruction, and impaired platelet production [28]. Consequently, elevated APRI may capture the combined presence of tissue injury and platelet dysregulation, both of which are biologically relevant features of severe systemic illness and organ dysfunction. Several studies support the prognostic relevance of the individual components of APRI in sepsis. Elevated AST levels and increased AST/ALT ratios have been associated with greater disease severity and mortality in patients with sepsis and septic shock. Schupp et al. demonstrated that the AST/ALT ratio independently predicted 30-day mortality and discriminated septic shock from sepsis, while a more recent multicenter study further supported its prognostic value for 28-day mortality [29,30]. Likewise, thrombocytopenia has long been recognized as an adverse prognostic feature in sepsis, with reduced platelet counts associated with increased mortality [31]. Importantly, the present findings extend previous observations by focusing specifically on the timing of mortality. Whereas prior sepsis studies have predominantly examined APRI in relation to sepsis-associated liver dysfunction, coagulopathy, or overall clinical outcomes, our results suggest that the association may be more pronounced for early mortality following ICU admission. However, this distinction should be interpreted cautiously because differences in study populations, outcome definitions, disease severity, and covariate adjustment limit direct comparisons across studies. These observations provide biological plausibility for the association between elevated APRI and mortality observed in the present study. By integrating AST and platelet count, APRI may capture complementary abnormalities occurring during severe critical illness. Nevertheless, APRI is a nonspecific composite index, and the present observational study cannot determine which underlying pathophysiological process is primarily responsible for its association with mortality. The proposed mechanisms should therefore be regarded as biologically plausible explanations supported by previous studies rather than causal pathways demonstrated by the present data.

The association between elevated APRI and adverse outcomes observed in the present study is consistent with an increasing body of evidence supporting the prognostic value of APRI in critically ill patients. Shi et al. reported that elevated APRI and other liver fibrosis indices were associated with increased mortality in patients with sepsis-induced coagulopathy, suggesting that liver-related biomarkers provide clinically relevant prognostic information in septic patients [32]. Similarly, Zhang et al., in a large cohort of 6334 patients with sepsis, demonstrated that higher APRI values were independently associated with the development of sepsis-associated liver dysfunction (SALD) and poorer clinical outcomes [18]. Although our study evaluated early (7-day) mortality rather than liver dysfunction, both studies support the concept that APRI reflects the severity of organ dysfunction occurring during sepsis. The prognostic significance of APRI has also been demonstrated in other critical illnesses. Yang et al. found that elevated APRI independently predicted mortality among patients with cardiogenic shock, while Wang et al. reported that APRI accurately predicted both 7-day and 28-day mortality in patients with heat stroke [20,22]. Despite differences in the underlying disease processes, these studies consistently suggest that APRI reflects systemic injury rather than liver dysfunction alone. Taken together, these findings support the possibility that elevated APRI may capture aspects of systemic injury occurring during critical illness rather than reflecting hepatic dysfunction alone. Nevertheless, given its nonspecific nature, APRI should be interpreted as a composite marker associated with critical illness severity rather than as an indicator of a specific underlying pathophysiological process.

Evidence from pediatric studies further strengthens the biological plausibility of our findings. Dou et al. were the first to demonstrate that APRI measured within 24 h of PICU admission independently predicted the development of sepsis-associated liver injury (SALI) in children and showed excellent diagnostic performance [19]. Similarly, Pantea et al. reported significantly higher APRI values in full-term newborns with systemic inflammatory response syndrome (SIRS), suggesting that APRI may also reflect broader systemic inflammatory processes beyond sepsis [33]. Although pediatric sepsis differs substantially from adult sepsis with respect to immune maturation, comorbidity profiles, and patterns of organ dysfunction [34,35], the consistent association between APRI and adverse outcomes across both age groups suggests that hepatic injury and platelet abnormalities represent fundamental components of the host response to severe infection rather than age-specific phenomena. The agreement between these pediatric observations and our findings supports the generalizability of APRI as a prognostic biomarker across different septic populations.

An important observation in the present study was the association between elevated admission APRI and reduced short-term survival demonstrated by Kaplan–Meier analysis. Notably, admission APRI showed a significant association with early mortality but not with overall in-hospital mortality, suggesting that this index primarily reflects the severity of the initial pathophysiological insult at the time of ICU admission. APRI is obtained at ICU admission and therefore primarily reflects the severity of the initial septic insult, including early hepatocellular injury, tissue hypoperfusion, systemic inflammation, and platelet consumption, processes that are considered major determinants of death during the acute phase of sepsis [36,37]. In contrast, mortality occurring later during hospitalization is influenced by numerous additional factors, including treatment response, secondary infections, evolving organ dysfunction, and pre-existing comorbidities [37,38,39,40], which may not captured by a single baseline APRI measurement. Consequently, admission APRI may be more effective for identifying patients at risk of early clinical deterioration than for predicting longer-term outcomes. However, several potential sources of confounding should also be considered when interpreting these findings. APRI is a nonspecific composite index, and elevations may arise from pre-existing chronic liver disease, acute hepatocellular injury, or thrombocytopenia unrelated to sepsis. In critically ill patients, platelet counts may also be influenced by disseminated intravascular coagulation, medications, hematological disorders, bleeding, or other causes of platelet consumption. Therefore, the observed association between APRI and early mortality cannot be attributed specifically to sepsis-induced hepatic injury or platelet consumption. In addition, although the multivariable analyses adjusted for age, sex, and major comorbidities, established measures of acute illness severity, such as SOFA and APACHE II scores, septic shock status, and vasopressor requirement, were not available for adjustment. Residual confounding by illness severity therefore remains possible, and elevated APRI may partly represent a marker of greater overall physiological derangement rather than an independent sepsis-specific pathway.

Beyond their individual prognostic value, laboratory-derived biomarkers such as APRI, NLR, SIRI, SII, and AISI may have increasing relevance in the era of artificial intelligence (AI) and machine learning (ML). Because these indices are inexpensive, standardized, routinely measured, and readily extracted from electronic health records, they represent attractive candidate features for AI-driven clinical decision support systems. Recent systematic reviews have demonstrated that machine learning models integrating routinely available laboratory parameters and clinical variables can improve early sepsis detection and prognostic prediction while emphasizing the growing importance of explainable AI to facilitate clinical implementation [41,42,43]. Consequently, laboratory-derived biomarkers are likely to provide the greatest clinical value when incorporated into multimodal prediction models alongside demographic characteristics, physiological measurements, and established severity scores, rather than being used as standalone predictors. Future studies should specifically determine whether adding APRI to established severity scores such as SOFA or APACHE II provides incremental prognostic information, including improvement in discrimination, calibration, and clinical net benefit.

## 5. Strengths and Limitations

The present study has several important strengths. To our knowledge, it is among the few studies evaluating admission APRI in relation to both 7-day and 28-day mortality among adult ICU patients with sepsis in a Saudi population. Nevertheless, several limitations should be acknowledged. The retrospective, single-center design may limit the generalizability of the findings and introduces the possibility of residual confounding. Importantly, established critical-illness severity scores, including SOFA and APACHE II, were not available in the retrospective dataset, and the clinical variables required for their reliable reconstruction were incomplete. Consequently, residual confounding by acute illness severity cannot be excluded, and the present findings cannot establish whether APRI provides prognostic information beyond established ICU severity scores. This is particularly relevant because AST and platelet count, the two components of APRI, may themselves be influenced by the severity of systemic illness and organ dysfunction. Furthermore, detailed information regarding pre-existing chronic liver disease and causes of thrombocytopenia was not consistently available for adjustment. As these conditions may directly influence AST and platelet count independently of the acute septic process, their potential confounding effects cannot be excluded. In addition, the moderate discriminatory performance for 7-day mortality indicates that APRI should not be considered a standalone prognostic tool.

Future research should focus on prospective, multicenter validation of APRI as an adjunctive prognostic marker in patients with sepsis. Studies should determine whether APRI provides incremental prognostic information beyond established severity scores such as SOFA and APACHE II, while accounting for potential confounders including underlying liver disease and causes of thrombocytopenia. Evaluation of serial APRI measurements may also clarify whether temporal changes provide additional prognostic information beyond a single admission value. Finally, the clinical value of incorporating APRI into multivariable prediction models should be assessed using measures of discrimination, calibration, and clinical net benefit before its routine use for risk stratification can be considered.

## 6. Conclusions

In summary, our findings demonstrate that elevated admission APRI is independently associated with early mortality, reduced short-term survival, and adverse clinical outcomes in ICU patients with sepsis. However, its modest discriminatory performance does not support its use as a standalone prognostic tool for clinical decision-making. APRI may instead represent a simple and readily available score that integrates hepatic injury and platelet dysregulation, two fundamental manifestations of sepsis-induced organ dysfunction. Further prospective studies incorporating established severity scores, such as SOFA or APACHE II, are required to determine whether APRI provides incremental prognostic information beyond conventional clinical assessment and improves model discrimination, calibration, or clinical utility.

## Figures and Tables

**Table 1:** Baseline Admission Characteristics According to Survival Status.

Variable	Survivors (*n* = 74)	Non-Survivors (*n* = 192)	*p* Value
Age (years)	62.0 (44.2–71.8)	64.0 (53.0–75.0)	0.082
BMI (kg/m^2^) ^a^	25.6 (21.3–29.8)	28.8 (24.0–33.7)	0.016
ICU LOS (days)	22.4 (14.6–57.9)	21.4 (11.2–39.7)	0.158
AST (U/L)	31.4 (20.2–96.8)	44.0 (22.6–106.0)	0.367
ALT (U/L)	23.5 (12.2–62.2)	26.0 (14.0–65.4)	0.496
Albumin (g/L)	29.7 (24.0–33.8)	26.2 (20.9–32.1)	0.005
Total Bilirubin (µmol/L)	10.4 (7.2–17.4)	11.5 (6.8–27.9)	0.344
CRP (mg/L) ^b^	111.0 (24.2–294.2)	125.0 (32.4–269.7)	0.831
Procalcitonin (ng/mL) ^c^	2.10 (0.35–9.88)	2.23 (0.56–5.52)	0.958
WBC (×10^9^/L)	13.2 (8.8–16.4)	10.8 (6.6–16.2)	0.079
RBC (×10^12^/L)	4.05 (3.40–4.80)	3.70 (3.00–4.40)	0.007
Hemoglobin (g/L)	110.0 (93.0–132.8)	102.0 (83.8–120.2)	0.018
Platelets (×10^9^/L)	235 (173.5–322.5)	208 (119.2–316.0)	0.174
Sex—Male, *n* (%)	44 (59.5%)	93 (48.4%)	0.107

Data are presented as median (Q1–Q3) unless otherwise stated. *p*-values were calculated using the Mann–Whitney U test for continuous variables and the Pearson Chi-square test for sex. ^a^ BMI available in 202 patients (survivors, *n* = 46; non-survivors, *n* = 156). ^b^ CRP available in 60 patients (survivors, *n* = 17; non-survivors, *n* = 43). ^c^ Procalcitonin available in 74 patients (survivors, *n* = 22; non-survivors, *n* = 52).

**Table 2:** Spearman Correlation Between Admission APRI and Clinical and Laboratory Parameters in ICU Patients with Sepsis.

Variable	Spearman’s r	*p* Value
White blood cell count (×10^9^/L)	−0.021	0.735
Neutrophils (×10^9^/L)	−0.034	0.613
Monocytes (×10^9^/L)	−0.046	0.487
Lymphocytes (×10^9^/L)	−0.008	0.902
Red blood cell count (×10^12^/L)	−0.040	0.518
Hematocrit (%)	0.000	0.994
Hemoglobin (g/L)	0.017	0.777
Albumin (g/L)	−0.127	0.038
Blood urea nitrogen (mmol/L)	0.128	0.037
Indirect bilirubin (µmol/L)	0.279	<0.001
Total bilirubin (µmol/L)	0.432	<0.001
C-reactive protein (mg/L)	−0.137	0.298
Procalcitonin (ng/mL)	0.134	0.254
Phosphorus (mmol/L)	0.169	0.006
Osmolality (mOsm/kg)	0.077	0.217
Sodium (mmol/L)	0.101	0.099
Total protein (g/L)	−0.100	0.105

Abbreviations: APRI, Aspartate Aminotransferase-to-Platelet Ratio Index; BUN, blood urea nitrogen; CRP, C-reactive protein; RBC, red blood cell; WBC, white blood cell.

**Table 3:** Prevalence of Elevated Admission APRI According to Mortality Status Among ICU Patients with Sepsis.

Mortality Group	APRI < 0.5, *n* (%)	APRI ≥ 0.5, *n* (%)	Pairwise Comparison with 7-Day Mortality (*p* Value)
7-day mortality	7 (21.9)	25 (78.1)	—
28-day mortality	35 (39.8)	53 (60.2)	0.0849
Late mortality	38 (52.8)	34 (47.2)	0.0049
Survivors	38 (51.4)	36 (48.6)	0.0055
Overall comparison			χ^2^ = 10.84, *p* = 0.0126
Trend analysis			χ^2^ = 8.449, *p* = 0.0037

**Abbreviations:** APRI, Aspartate Aminotransferase-to-Platelet Ratio Index.

**Table 4:** Unadjusted and Adjusted Logistic Regression Analyses of the Association Between Admission APRI and 7-Day Mortality Among ICU Patients with Sepsis.

Variable	Statistic	Unadjusted	Model 1 †	Model 2 ‡
APRI	β (95% CI)	1.009	1.009	1.018
		(0.460 to 1.583)	(0.444 to 1.599)	(0.449 to 1.611)
	OR (95% CI) *p* value	2.74 (1.58–4.87)	2.74 (1.56–4.95)	2.77 (1.57–5.01)
		0.0003	0.0005	0.0004
Age	β (95% CI)	—	0.006	0.006
			(−0.016 to 0.029)	(−0.016 to 0.029)
	OR (95% CI) *p* value	—	1.01 (0.98–1.03)	1.01 (0.98–1.03)
			0.584	0.585
Sex	β (95% CI)	—	0.371	0.369
			(−0.403 to 1.177)	(−0.406 to 1.177)
	OR (95% CI) *p* value	—	1.45 (0.67–3.25)	1.45 (0.67–3.24)
			0.349	0.353
Comorbidity	β (95% CI)	—	—	0.361
				(−0.503 to 1.171)
	OR (95% CI) *p* value	—	—	1.43 (0.60–3.23) 0.401

Abbreviations: β, logistic regression coefficient; OR, odds ratio; CI, confidence interval; APRI, Aspartate Aminotransferase-to-Platelet Ratio Index. † Model 1: Adjusted for age and sex. ‡ Model 2: Adjusted for age, sex, and the presence of one or more major comorbidities.

**Table 5:** Receiver Operating Characteristic (ROC) Curve Analysis of Admission APRI for 7-Day and 28-Day Mortality in ICU Patients with Sepsis.

Outcome	AUC (95% CI)	*p* Value	Optimal Cut-Off	Sensitivity (%)	Specificity (%)
7-day mortality	0.687	<0.001	APRI > 1.015	68.4	65.6
	(0.580–0.793)				
28-day mortality	0.623	<0.001	APRI > 0.627	62.3	60.8
	(0.555–0.690)				

Abbreviations: AUC, area under the receiver operating characteristic curve; APRI, Aspartate Aminotransferase-to-Platelet Ratio Index; CI, confidence interval; LR+, positive likelihood ratio.

**Table 6:** Multivariable Cox Proportional Hazards Regression Analysis for 7-Day and 28-Day Mortality Among ICU Patients with Sepsis.

Variable	7-Day Mortality		28-Day Mortality	
	HR (95% CI)	*p* Value	HR (95% CI)	*p* Value
APRI	2.59 (1.55–4.32)	<0.001	1.91 (1.44–2.54)	<0.001
Age	1.01 (0.99–1.03)	0.398	1.02 (1.01–1.03)	0.003
Sex	1.46 (0.72–3.10)	0.296	1.22 (0.85–1.75)	0.290
Comorbidities	1.34 (0.61–2.76)	0.450	1.34 (0.90–2.00)	0.149

## Data Availability

The datasets generated and/or analyzed during the current study are available from the corresponding author upon reasonable request, subject to approval by the hosting institution.
